# Patient experience and repeatability of measurements made with the Pentacam HR in patients with keratoconus

**DOI:** 10.1186/s12886-023-02930-4

**Published:** 2023-05-08

**Authors:** Ingemar Gustafsson, Dimitrios Bizios, Anders Ivarsen, Jesper Ø Hjortdal

**Affiliations:** 1 Department of Ophthalmology, Skåne University Hospital, Department of Clinical Sciences, Lund University, Lund, Sweden; 2 Department of Ophthalmology, Aarhus University Hospital, Aarhus, Denmark

**Keywords:** Keratoconus, Tomography, Repeatability, Pentacam HR

## Abstract

**Background:**

To investigate whether the repeatability of measurements with the Pentacam HR in patients with keratoconus is improved by patients gaining more experience of the measurement situation. Such an improvement could enhance the accuracy with which progressive keratoconus can be detected.

**Methods:**

Four replicate measurements were performed on Day 0 and on Day 3. Parameters commonly used in the diagnosis of progressive keratoconus were included in the analysis, namely the flattest central keratometry value (K1), the steepest central keratometry value (K2), the maximum keratometry value (Kmax), and the parameters A, B and C from the Belin ABCD Progression Display. In addition, quality parameters used by the Pentacam HR to assess the quality of the measurements were included, namely the analysed area (front + back), 3D (front + back), XY, Z, and eye movements.

**Results:**

Neither the diagnostic parameters nor the quality parameters showed any statistically significant improvement on Day 3 compared to Day 0. The quality parameter “eye movements” deteriorated significantly with increasing Kmax.

**Conclusion:**

Gaining experience of the measurement situation did not increase the accuracy of the measurements. Further investigations should be performed to determine whether the increasing number of eye movements with increasing disease severity has a negative effect on the repeatability of the measurements.

**Supplementary Information:**

The online version contains supplementary material available at 10.1186/s12886-023-02930-4.

## Introduction

Corneal crosslinking (CXL) is used worldwide to arrest the progression of keratoconus disease [1]. Pre-clinical investigations have shown that CXL stiffens the cornea [2] while clinical investigations have indicated its clinical efficacy in halting keratoconus disease progression [3], also in the long-term perspective [4]. Furthermore, it has been suggested that CXL reduces the need for corneal transplantation [5]. The most common indication for CXL is progressive keratoconus [6], however, children and adolescents are usually referred to CXL upon the diagnosis of keratoconus as the risk of progression is high in younger patients [3, 7]. Progressive keratoconus is commonly assessed by subjective factors such as medical history and visual acuity [8], in addition to objective parameters measured by corneal tomography. The most commonly used corneal tomographer in scientific investigations is the Pentacam HR (Oculus Optikgeräte, GmbH, Wetzlar, Germany) [1]. Less is known about the equipment used in clinical practice, but a recent survey of northern European countries suggested that the Pentacam HR was the most commonly used tomographer [7].

In terms of objective parameters measured by the Pentacam HR, the most frequently used parameter in scientific investigations is the maximum keratometry value (Kmax) [6, 9–11], and sometimes also the steepest central keratometry value (K2) [3]. From the clinical perspective, Kmax has been suggested to be the most important parameter, followed by the parameters A, B and C from the Belin ABCD Progression Display in the Pentacam HR [7]. We therefore included these parameters in the analysis, together with the flattest central keratometry value (K1), as K1 and K2 are commonly used for the objective assessment of astigmatism. All the parameters included reflect the opinions expressed regarding the assessment of progressive keratoconus in the Global Consensus on Keratoconus and Ectatic Diseases [12].

It is of the utmost importance to diagnose progressive keratoconus accurately for timely referral to CXL, and for the reliable recruitment of patients with truly progressive keratoconus in clinical trials [13, 14]. As there is no gold standard for assessing progressive keratoconus, diagnosis must be based on a reliable calculation of the measurement accuracy, i.e., the repeatability of the measurements made with the equipment used for examination [15]. Numerous articles have been published on the general repeatability of measurements using the Pentacam HR in patients with keratoconus and healthy controls [16–18]. Furthermore, specific factors that can affect the repeatability have been investigated, such as inter-observer effects [19], the effects of disease severity[20], the number of replicate measurements and inter-day effects [13]. However, an aspect that merits further evaluation is the possible effect of the patient gaining experience of the measurement situation. In other areas of ophthalmology, such as perimetry in glaucoma, it has been demonstrated that the results improve as patient experience of the measurement situation increases [21]. In a previous analysis of the inter-day repeatability of measurements using the Pentacam HR in patients with keratoconus, the keratometric parameters were found to show better repeatability on the second measurement occasion (Day 3 compared to Day 0) [13]. It is thus of interest to determine whether the measurement accuracy can be improved by increasing the patient’s experience of the measurement situation.

## Subjects and methods

The study was conducted at the Department of Ophthalmology at Skåne University Hospital, Lund, Sweden, according to the tenets of the declaration of Helsinki. All participants were given written information on the study, and written consent was obtained The Swedish Ethical Review Authority in Lund, Sweden, approved the studies (No. 2015/373).

The following diagnostic parameters were included as these are frequently used in scientific and clinical practice [7].

- The flattest central keratometry value in a 15 degree ring around the apex (K1).
- The steepest central keratometry value in a 15 degree ring around the apex (K2).
- The maximum keratometry value (Kmax).
- The anterior curvature of the 3 mm zone over the thinnest point of the cornea (A), the posterior curvature of the 3 mm zone over the thinnest point of the cornea (B) and the thickness at the thinnest point of the cornea (C), from the Belin ABCD Progression Display (22).

The following quality parameters were included as these could be dependent, or partially dependent, on the patient or the operator.

- Analysed area (front): This parameter ensures that the measured area is sufficient. The measurement of this parameter can be affected if the patient’s eye is not sufficiently open, by blinking, or when the patient has long eye lashes.
- Analysed area (back): as above, but for the posterior surface.
- 3D (front and back): This parameter ensures correct 3D modelling of the anterior and posterior surfaces of the cornea. Excessive blinking, loss of fixation or insufficient opening of the eyes affects this parameter negatively.
- XY and Z: These parameters describe whether the operator of the Pentacam HR has moved the X, Y, Z base slide at the moment the measurement started. These parameters were included to account for effects of the operator on the measurements.
- Eye movements: this parameter describes excessive eye movements due to loss of fixation.

The following quality parameters were excluded as these were deemed not to be affected by better patient compliance.

- Valid data: This parameter ensures that a sufficient number of valid data points are found. Factors that can affect this parameter are not blinking before the measurement and the room illumination. As all the patients were instructed to blink before the measurement, and as the illumination in the room was the same on all measurement occasions, this parameter was excluded.
- Lost segments and lost segments continuous. These parameters are binary, (0 = Approved, 1 = Not approved).

### Participant enrolment [13]

Patients with keratoconus fulfilling the inclusion criteria described below were enrolled consecutively. The inclusion criteria were: keratoconus Stage ≤ 2 [23] with no history of, and no current signs of other ocular pathology, including ocular surface disease and external diseases such as dry eyes and atopy. Only subjects who had not previously undergone ocular surgery and who were aged ≥ 18 years were recruited. Pregnant and breastfeeding women were also excluded. Contact lens wear was discontinued at least 2 weeks before the measurements were made. Patients with Stage 3–4 keratoconus were excluded as the purpose was to study those with less advanced disease. Keratoconus was diagnosed clinically and by examination using the Pentacam HR. The sagittal curvature pattern, posterior and anterior elevation maps, and corneal thickness pattern were assessed, in addition to information from the Belin-Ambrosio Enhanced Ectasia Display.

Twenty-five patients were enrolled. If two eyes were eligible for inclusion, both were examined (see *Examination* below). Computerised randomisation was performed in patients where both eyes met the inclusion criteria to select one eye for inclusion in the study. Twenty-two participants were male and three female, and the mean age of the group was 27 years (range 21–45 years). Twelve right and thirteen left eyes were included.

### Equipment [13]

The Pentacam HR is a Scheimpflug-based tomographic system (Pentacam HR, version 1.20r10, Oculus Optikgeräte GmbH, Wetzlar, Germany). The technical features of this system have been described elsewhere [16]. The default setting of 25 pictures per second was used.

### Examination [13]

Four replicate measurements were made on two separate occasions (Day 0 and Day 3) by the same examiner (IG). Subjects were instructed to blink between measurements, but not to lean back. Measurements were made during normal working hours. Only examinations deemed “OK” by the Pentacam HR were accepted. The right eye was examined first, then the left, if both eyes were eligible for inclusion. This reflects normal clinical practice, where both the patient’s eyes are usually examined. When recruitment to the study was complete, computerised randomisation was performed to select one eye per subject.

### Statistical methods and calculations [13]

IBM SPSS Statistics 22 for Windows (IBM Corporation, Armonk, NY, USA) and SAS Enterprise Guide 6.1 for Windows (SAS Institute Inc., Cary, NC, USA) were used for statistical analyses. A p-value below 0.05 was considered significant. Descriptive statistics are given as subject mean, standard deviation (SD), and minimum and maximum values. Repeatability was assessed by calculating the within-subject standard deviation (S_w_), precision, repeatability coefficient, intra-class correlation (ICC) and coefficient of variation (CV%) with associated confidence intervals (CIs) [15, 24, 25]. Kendall’s Tau-b was used to assess the relationship between the mean and SD, and natural-logarithm-transformed data were analysed when appropriate. Differences between coefficients of variation were assessed using a regression test [26]. The Wilcoxon signed ranks test was used for comparisons of the means of the quality parameters on Day 0 and Day 3. A professional medical statistician was consulted and performed the analysis.

## Results

### Diagnostic parameters

Descriptive statistics and the repeatability of the measurements of the diagnostic parameters on Day 0 and Day 3 are presented in Table 1. The anterior keratometric parameters (K1, K2, Kmax and A) all showed better repeatability on Day 3. Parameter B showed a slightly poorer repeatability, while that of parameter C was unchanged. Kmax appeared to show the greatest improvement in the repeatability of the measurements, with a 0.18 D improvement on Day 3 (Day 0, 0.70 D and Day 3, 0.52 D); the 95% CIs on Day 0 and Day 3 barely overlapped. However, a regression test comparing the coefficients of variation on Day 0 and Day 3 did not result in any statistically significant difference in Kmax, or for any of the other parameters.

**Table 1:** Descriptive statistics and repeatability of the measurements on Day 0 and Day 3 in subjects with keratoconus

	Day	Mean (SD)	Min–Max	S_w_ (95% CI)	CV%	Repeatability (95% CI)	Regression test, p-value*
K1 (D)							
	0	43.6 (1.8)	(40.7–47.5)	0.15 (0.13–0.18)	0.35	0.42 (0.35–0.49)	
	3	43.6 (1.8)	(40.6–47.2)	0.14 (0.12–0.16)	0.32	0.38 (0.32–0.44)	0.5
K2 (D)							
	0	46.0 (2.8)	(42.8–56.0)	0.24 (0.20–0.28)	0.54^a^	0.67 (0.56–0.78)	
	3	46.0 (2.7)	(42.8–55.7)	0.19 (0.16–0.22)	0.41	0.52 (0.44–0.61)	0.12
Kmax (D)							
	0	50.3 (4.8)	(44.5–65.4)	0.25 (0.21–0.29)	0.46^a^	0.70 (0.59–0.81)	
	3	50.2 (4.7)	(44.4–64.7)	0.19 (0.16–0.22)	0.35^a^	0.52 (0.44–0.61)	0.70
A (mm)							
	0	7.18 (0.48)	(6.34–7.99)	0.054 (0.045–0.062)	0.78^a^	0.15 (0.12–0.17)	
	3	7.17 (0.49)	(6.34–7.90)	0.045 (0.037–0.052)	0.62	0.12 (0.10–0.14)	0.63
B (mm)							
	0	5.53 (0.51)	(4.68–6.45)	0.048 (0.040–0.056)	0.87	0.13 (0.11–0.15)	
	3	5.52 (0.52)	(4.58–6.43)	0.054 (0.046–0.063)	0.99	0.15 (0.13–0.17)	0.75
C (µm)							
	0	492.6 (35.0)	(442.8–560.3)	3.85 (3.23–4.46)	0.78	10.7 (8.96–12.4)	
	3	492.8 (35.3)	(437.5–561.3)	3.84 (3.23–4.46)	0.78	10.6 (8.94–12.4)	0.73

K1: flattest central keratometry value, K2: steepest central keratometry value, Kmax: maximum keratometry value, A: anterior curvature of the 3 mm zone over the thinnest point, B: posterior curvature of the 3 mm zone over the thinnest point and C: thickness of the thinnest point on the cornea

^a^Calculated using natural logarithm transformation. *A p-value below 0.05 was considered significant

### Quality parameters

Descriptive statistics of the quality parameters and the results of the Wilcoxon signed ranks test between Day 0 and Day 3 are presented in Table 2. All the quality parameters had high standard deviations, and the results of the Wilcoxon signed ranks test must thus be interpreted with caution. Apart from the parameter Z, no statistically significant differences were found between Day 0 and Day 3. The value of Z was significantly lower on Day 0 (177) than on Day 3 (220), suggesting that the operator moved the base slide of the instrument more at the moment when the measurement started. However, the high SD (88 on Day 0 and 100 on Day 3) makes the outcome of the Wilcoxon signed ranks test highly unreliable and difficult to interpret.

**Table 2:** Values of the quality parameters (mean) with associated standard deviations (SD) on Day 0 and Day 3 together with the results of the paired-samples tests for Day 0 and Day 3

	Day	Mean (SD)	p-value
XY mean	0	240 (87)	
	3	232 (86)	0.699
Z mean	0	177 (88)	
	3	220 (100)	0.028
Eye mov	0	91 (22)	
	3	92 (19)	0.772
Area (front)	0	79 (5.3)	
	3	79 (5.2)	0.946
Area (back)	0	65.5 (4.0)	
	3	65.5 (4.1)	0.935
3D (front)	0	1.5 (0.60)	
	3	1.4 (0.40)	0.414
3D (back)	0	7.7 (2.0)	
	3	7.6 (1.8)	0.620

XY and Z: quantify movements of the base slide, Eye Mov: quantifies excessive movements, Area (front and back): the measured anterior and posterior surfaces, 3D (front and back) the correct modelling of the anterior and posterior surfaces. *A p-value below 0.05 was considered significant

The high variation in the quality parameters is illustrated in the figures given in the Supplementary Information: Z (Fig. S1), XY (SI 2), Eye movements (SI 3), 3D front (SI 4), 3D back (SI 5), Area front (SI 6) and Area back (SI 7).

**Fig. 1:**
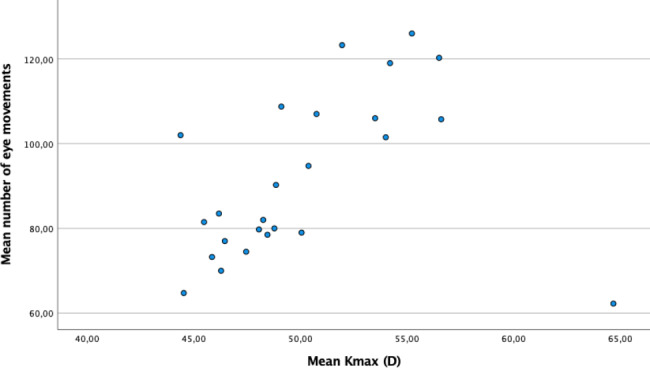
The mean value of Kmax for each patient plotted against the mean value of the number of eye movements

Increasing keratoconus disease severity can lead to increasing difficulties in focusing during the measurements, thus increasing the number of eye movements. A correlation test was thus performed between Kmax and the eye movement parameter, revealing a highly significant association between Kmax and the number of eye movements (Kendall’s Tau–B, 0.440, p = 0.002) (Fig. 1).

## Discussion

The results of this investigation suggest that there is no need to perform measurements on more than one occasion to increase the patient’s experience of the measurement situation for the purpose of increasing the accuracy with which progressive keratoconus can be detected. Although the repeatability of the measurements of keratometric parameters was better on Day 3, no statistically significant differences were found. Furthermore, the quality parameters given by the Pentacam HR, which are used to assess the quality of the measurements, showed no statistically significant improvement on Day 3 compared to Day 0. The variation of the different quality parameters appears to be random, and not associated with a specific replicate in the chain of measurements, as could be suspected. On the one hand, the initial measurements could be associated with poorer quality scores as the patient is less experienced, and then improve with increasing experience. On the other hand, performing repeated measurements could also tax the patient’s attention, leading to reduced compliance during the measurement procedure.

In previous investigations we have shown the importance of using a mean of replicates when assessing progressive keratoconus, as this increases the accuracy of the diagnosis compared to the use of single measurements [13, 27]. Therefore, it is important to understand that this study demonstrates that up to four replicates can be performed on each occasion without affecting the repeatability of the measurements on each occasion.

In other areas of ophthalmology, such as perimetry in glaucoma, it has been shown that increasing experience in testing leads to better test reliability [21]. The perimetric examination is highly dependent on the individual’s responses, and several factors can affect these responses through learning or experiencing the test procedure. One such factor is the attention span needed to maintain a steady gaze and to give a timely response to each light stimulus during the examination, which often lasts from two to several minutes. The reason why measurements with the Pentacam HR do not improve with increasing patient experience could be the short measurement period, which only lasts about 2 s. However, keeping the gaze fixed on the red spot for 2 s may be challenging for patients with keratoconus. The irregular astigmatism deforms the shape of the red fixation spot, which can provoke eye movements in search of an optimal gaze position. In fact, this study revealed a strong statistically significant association between increasing number of eye movements and increasing values of Kmax. It remains to be elucidated whether the association is a contributing or causative factor to the well-known association between deteriorating repeatability of the measurements and increasing keratoconus disease severity, as we have demonstrated in Pentacam HR measurements in both an intra-day [20] and inter-day setting [13].

The strength of this study is that the measurements were performed by the same examiner, thus avoiding possible inter-examiner effects [19]. In addition, measurements were performed during normal working hours, reducing the likelihood of diurnal effects [28–30]. Furthermore, the illumination of the room was the same on all measurement occasions. A limitation is that only subjects with less advanced keratoconus were recruited, therefore, the results of this study are only applicable to this group. A further limitation is that the results of the inter-day Wilcoxon signed ranks test of the XY and Z parameters must be interpreted with caution. The high standard deviation of these parameters makes the results unreliable. A prospective evaluation would, however, require a large number of participants to provide adequate power for the analysis of such data. Future investigations should also be carried out to determine the inter-day repeatability when using optical coherence tomography equipment for the diagnosis of progressive keratoconus.

In summary, the results of this study show that the repeatability and the quality of the measurements with the Pentacam HR do not improve with increasing experience of the measurement situation in patients with less advanced keratoconus. However, it was found that the number of eye movements during the measurement procedure increased with increasing keratoconus disease severity. As it is well-known that the repeatability deteriorates with increasing disease severity, it would be of considerable interest to investigate whether the increasing number of eye movements contributes to the deterioration in the repeatability of the measurements. Accounting for this could lead to increased measurement precision in these subjects, which would be of considerable clinical value.

## Electronic supplementary material

Below is the link to the electronic supplementary material.

Supplementary Material 1

Supplementary Material 2

Supplementary Material 3

Supplementary Material 4

Supplementary Material 5

Supplementary Material 6

Supplementary Material 7

Supplementary Material 8

Supplementary Material 9

## Data Availability

The datasets with all data are available as supplementary information.

## List of Abbreviations

CI: Confidence Interval
CV%: Coefficient of variation
CXL: Corneal Cross-linking
SD: Standard Deviation
Sw: Within-subject standard deviation
