# Supplementary material for: Patient experience and repeatability of measurements made with the Pentacam HR in patients with keratoconus
Source: BMC Ophthalmol. 2023 May 8;23:201. doi: 10.1186/s12886-023-02930-4 (PMC10165742; doi:10.1186/s12886-023-02930-4)

Supplementary information 4. Plots showing the variation in the values of the 3D font parameter on the different measurement occasions. The 25 keratoconus patients are colour coded. A vertical reference line is inserted between Day 0 and Day 3.


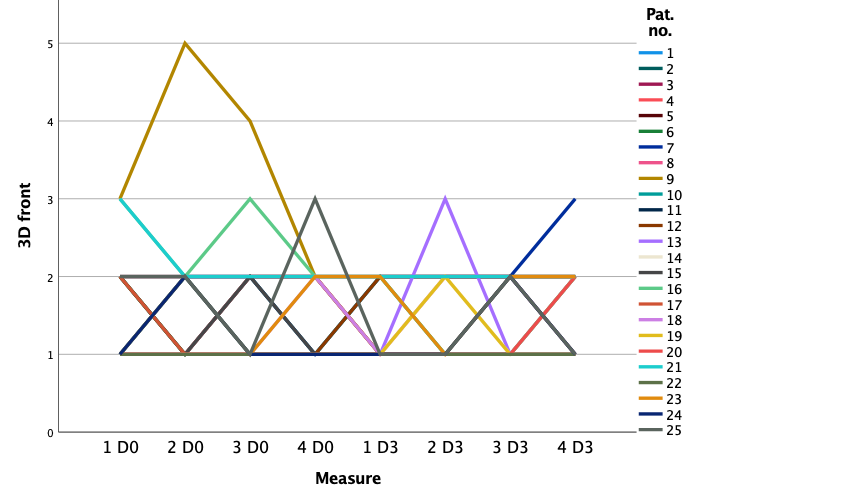

Supplement: Supplementary file 6 — Supplementary Material 6 [file 12886_2023_2930_MOESM6_ESM.docx]
